# Control of source fertility on the eruptive activity of Piton de la Fournaise volcano, La Réunion

**DOI:** 10.1038/s41598-018-32809-0

**Published:** 2018-09-27

**Authors:** I. Vlastélic, A. Di Muro, P. Bachèlery, L. Gurioli, D. Auclair, A. Gannoun

**Affiliations:** 1 Université Clermont Auvergne, CNRS, IRD, OPGC, Laboratoire Magmas et Volcans, F-63000 Clermont, Ferrand France; 2 Observatoire Volcanologique du Piton de la Fournaise, Institut de Physique du Globe, Sorbonne Paris-Cité, CNRS UMR 7154, Université Paris Diderot, Paris, France

## Abstract

The eruptive activity of basaltic hotspot volcanoes displays major fluctuations on times scales of years to decades. Theses fluctuations are thought to reflect changes in the rate of mantle melt supply. However, the crustal filter generally masks the mantle processes involved. Here, we show that the cyclic and generally increasing activity of the Piton de la Fournaise volcano (La Réunion) since the mid 20th century is tightly linked to the fertility of its source, as recorded by ^87^Sr/^86^Sr and incompatible trace elements ratios of lavas. We identify a twofold control of source fertility on eruptive activity: melt extraction from fertile, incompatible element-enriched veins initiates decadal-scale eruptive sequences, so that vein distribution in the plume source directly controls the cyclic activity. Indirectly, reactive flow of enriched melts increases mantle porosity and promotes melts extraction from the peridotite matrix. This process is thought to have caused a fourfold increase in magma supply between 1998 and 2014 at Piton de la Fournaise, and could also explain magma surges at other frequently active hotspot volcanoes, such as Kilauea, Hawaii. The short-term eruptive activity of hotspot volcanoes appears to be ultimately linked to the distribution and size of lithological heterogeneities in mantle plumes.

## Introduction

Ocean island basaltic shield volcanoes, such as Kilauea (Hawaii) or Piton de la Fournaise (La Réunion) are directly connected to zones of persistent melting in the Earth’s mantle^1,2^. Over the last century, their rates of lava production have varied by a factor of two to ten over timescales of years to decades^3,4^. Such variations are difficult to explain solely by shallow magma chamber processes. Indeed they result partly from variations in the rate or timing of mantle melt supply. Temporal-compositional variations of erupted lavas provide robust clues to follow the rapid processes that take place in the volcano sources^5,6^. For instance, the increasing eruptive activity of Kilauea volcano since 1924 correlates with source- and melting-related fluctuations of incompatible trace element and isotope ratios^7,8^. Summit lava lakes and long-lived rift-zone eruptions that occurred since 1950 can be related to the input of new, mantle-derived magma batches^9–11^. On the other side of the planet, Piton de la Fournaise volcano has produced numerous short-lived eruptions over the last century, with no eruption lasting more than six months. Since the early 20^th^ century, eruptions cluster into 12–24 years long sequences followed by three-to-six year-long reposes which, together, define the eruptive cycles^4,12^. These decadal cycles could reflect the time needed to refill the plumbing system with mantle melts^12^. Such processes at Piton de la Fournaise are not readily seen in most geochemical and geophysical records, which are overprinted by higher frequency (<1 year) processes affecting the shallow magma reservoir^13,14^. This paper reports a high-resolution temporal record of lava Sr isotope composition over the most recent, well-characterized 1942–2017 period. The record shows that the cyclic and overall increasing eruptive activity of Piton de la Fournaise is tightly related to the melting of fertile, incompatible element-enriched mantle, and thus ultimately, to the distribution of mantle heterogeneities in the mantle plume source.

## Results

### Sr isotope record of eruptive cycles

**Figure 1:**
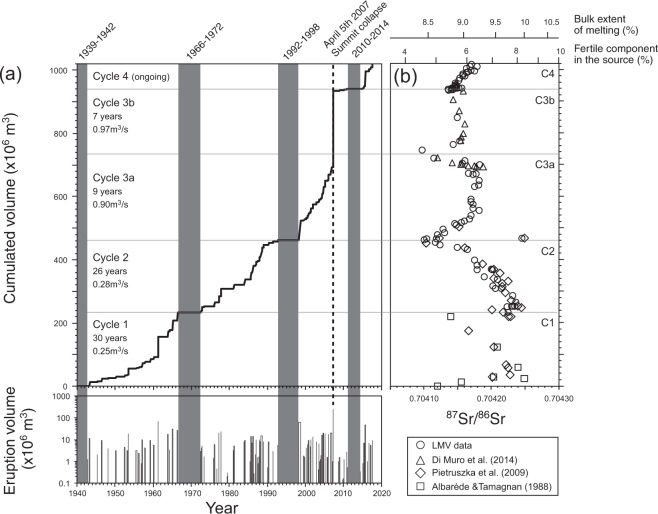
Bulk volume and ^87^Sr/^86^Sr of lava produced by Piton de la Fournaise volcano between 1942 and 2017. (**a**) Volumes of individual eruptions (lower panel) and cumulated erupted volume (upper panel) (Supplementary Table S1). Uncertainty of volumes of recent eruptions is estimated to be ca. 30%^14^ but is probably larger for cycle 1 eruptions. Vertical grey bands indicate inactivity periods of 3 years or longer, which are used to define major eruptive cycles. The dashed line indicates the April 5^th^ 2007 summit collapse, which separates cycle 3a from cycle 3b. (**b**) ^87^Sr/^86^Sr ratio plotted versus cumulated volume of lava. C1 to C4 refer to the eruptive cycles 1 to 4. Measurement error on ^87^Sr/^86^Sr is within data point. Data source: Albarède and Tamagnan^6^ and Bosch *et al*.^47^ for the 1942–1948 period (squares), Pietruszka *et al*.^17^ for the 1950–1998 period (diamonds), Di Muro *et al*.^48^ (triangles), and Laboratoire Magmas et Volcans (LMV) data (Vlastelic *et al*.^21^, Schiano *et al*.^23^ and this study, all shown by circles) for the 1972–2017 period (Supplementary Table S2). The fraction of fertile component in the source and the bulk extent of melting are inferred from the modelling of ^87^Sr/^86^Sr melt extraction trajectories, and shown here assuming a highly fertile (G2- type) pyroxenite (see methods). Slightly higher fractions of fertile material (8.7–4.8%), and lower and more uniform bulk extents of melting (7.4–7.0%) are obtained with a moderately fertile pyroxenite (KG-1 type).

The Piton de la Fournaise had 109 eruptions that produced 1.02 km^3^ of lava from 1942 to 2017 (Supplementary Table S1 and Fig. 1a). Eruptions clustered into decadal-scale sequences followed by 3+ years periods of quiescence, together forming eruptive cycles (hereafter defined by the beginning and the end of an eruption sequence, and the end of the following period of quiescence). Cycle 1 (1942–1966; 1972) and cycle 2 (1972–1992; 1998) produced similar volumes of lava (~234 and 229 × 10^6^ m^3^, respectively). Cycle 3 (1998–2010; 2014) produced a two times larger volume (478 × 10^6^ m^3^), including 240 × 10^6^ m^3^ of lava emitted during the withdrawal of the magma chamber in April 2007^15^. The ongoing eruption sequence (2014–2017), thought to start the new cycle 4, produced so far ~78 × 10^6^ m^3^ of lava. New major-trace element and isotope (Sr, Pb) data are reported for 60 samples (see methods), in order to complement and extend the existing geochemical record (Supplementary Table S2). Lava composition does not define a temporal trend at the scale of the century, but displays systematic short-term cyclic fluctuations^6,16^. The new high-resolution temporal record shows that the small ^87^Sr/^86^Sr variations (0.704097–0.704249) tightly correlate with the eruptive cycles (Fig. 1b). Within each cycle ^87^Sr/^86^Sr displays a similar and systematic evolution, including an early, rapid increase followed by a decrease towards the end of the cycle. The temporal trends of ^87^Sr/^86^Sr decrease within cycles 1 and 2 were previously identified by Pietruszka *et al*.^17^. Within the voluminous cycle 3 (1998–2014), Sr isotopes clearly define two internal cycles that correspond to the time-periods before (cycle 3a, 257 × 10^6^ m^3^) and after (cycle 3b, 221 × 10^6^ m^3^) the April 5^th^ 2007 summit collapse. Unlike other cycles, the two cycles 3a and 3b are not separated by a period of inactivity, but are linked by a major effusive paroxysm. During the best monitored last three cycles (3a, 3b, and 4), ^87^Sr/^86^Sr increased mostly during the voluminous eruptions of March 1998, April 2007 and August 2015 that initiated the cycles. Such early increase of ^87^Sr/^86^Sr might be related to the flushing of late-stage magma from previous cycle by new mantle-derived magmas. Seismic data provides evidence for such deep mantle inputs at the onset of cycles 3a and 4^18–20^. Major elements (MgO content, or CaO/Al_2_O_3_) also record the input of less differentiated magma during the major eruptions initiating the eruption cycles, but they poorly correlate with Sr isotopes beyond these initial eruptions. Lead isotopes do not correlate with Sr in a simple fashion^21^, whereas Nd isotopes vary barely outside analytical error^16^.

### Control of mantle heterogeneities on eruptive cycles

The small variations of Sr isotope compositions of Piton de la Fournaise lavas were first ascribed to assimilation of seawater-altered rocks from the oceanic basement^22^. However, subsequent studies based on Th and Os isotopes showed that crustal contamination is small and limited to few samples^17,23^. The correlation between ^87^Sr/^86^Sr and ratios of highly to moderately incompatible trace elements (Supplementary Fig. S3), previously observed by Pietruszka, *et al*.^17^, indicates that Sr isotope variations dominantly record long-lived chemical heterogeneities of the Reunion mantle plume^16^. Pietruszka, *et al*.^17^ argued that the greater enrichment of highly incompatible elements in high-^87^Sr/^86^Sr lavas is consistent with a more fertile source assuming that the melt fraction does not vary widely. The authors also suggested that the successive temporal trends of decreasing ^87^Sr/^86^Sr that occurred between 1953 and 1991 reflect individual melting events in the mantle, during which melts are progressively extracted from a less fertile, incompatible-element depleted source. Such a model might also explain the tight correlation between Sr isotope cycles and eruptive cycles shown on Fig. 1. The small variations of major element content of historical lavas, beyond olivine fractionation, and their poor correlation with ^87^Sr/^86^Sr provide few constraints on the lithological heterogeneity of the mantle source. The occurrence of cm-thick layers of clinopyroxenite within entrained dunite nodules^24^ nevertheless suggests the occurrence of calcic melts in the volcano source. For the purpose of modelling melt extraction trajectories, it is convenient to assume that the fertile and refractory sources are pyroxenitic and peridotitic in composition, respectively, although the high Mg content of most olivine crystals (Fo_80–88_)^25^ indicates that the bulk source is more or less fertile peridotite. The origin of the fertile lithology is uncertain and could be related to the small amount of oceanic crust recycled in the Reunion plume^23,26,27^.

Evolution of ^87^Sr/^86^Sr during melting of low-solidus-temperature pyroxenite, embedded in refractory peridotite is modelled following Stracke and Bourdon^28^ using input parameters relevant to the Reunion plume (see methods). Assuming first a highly fertile pyroxenite similar in composition to oceanic crust (G2 type), we find that the fraction of fertile material in the lava source decreases from 6–8.4% at the beginning of a cycle (after the complete flushing of late-stage magma from previous cycle) to 4.5% at the end (Fig. 1b). Extraction of melts from a progressively less fertile source during an eruption sequence results in a decrease of the bulk extent of melting from 10 to 8.4%. Considering a moderately fertile pyroxenite (KG-1 type) that starts melting just before the peridotite yields slightly higher fractions of fertile material in the source (8.7–4.8%), and lower and more uniform bulk extents of melting (7.4–7.0%). In both cases, model results (Fig. 1b) suggest that a threshold concentration of fertile pyroxenite (ca. 6%) in the source is needed to initiate an eruptive sequence.

The decrease of ^87^Sr/^86^Sr during an eruption sequence indicates that eruptions are increasingly fed by melts originating from the refractory peridotite matrix (Fig. 1b). This might have a general applicability as many intraplate basaltic volcanoes worldwide, such as Kilauea (Hawaii), Lanzarote (Canary) and Pisgah crater (South California), similarly produce increasingly depleted lavas during 5–20 yrs eruption sequences^29^. During an eruptive cycle of Piton de la Fournaise, as during the voluminous Kilauea’s eruptions^30^, melts must be drained from an increasing volume of mantle in order to sustain the flow of melt to the surface. By combining lava volume data with pyroxenite and peridotite mass fractions inferred from Sr isotope mass balance (see methods), we estimate that the volume of refractory mantle (1.0–2.4 km^3^) sampled over an eruption sequence is 14 to 17 times that of fertile mantle (0.06–0.16 km^3^). Thus, the distribution of small fertile veins in the upwelling plume may enable melt extraction from large refractory peridotite regions and control the eruptive cycles of Piton de la Fournaise. It is suggested that cycles 1, 2 and 3b ended with a period of inactivity because the distance between major fertile veins was larger than the size of the mantle region from which melts can be continuously extracted. Conversely, the absence of inactivity period between cycles 3a and 3b suggests that a new fertile vein was tapped before the exhaustion of melts from cycle 3a. The high frequency of the eruptive cycles (every 7 to 30 years) remains difficult to explain by the passage of small fertile heterogeneities through the melting zone because the plume upward velocity (5–9 cm/yr)^22^ is too slow. Instead, it is consistent with short-term changes in the location from which melt is extracted, as proposed for Kilauea^10^. These changes might result from tapping of veins distributed in the three dimensions of the melting region.

### Melt channelization as a consequence of source fertility

**Figure 2:**
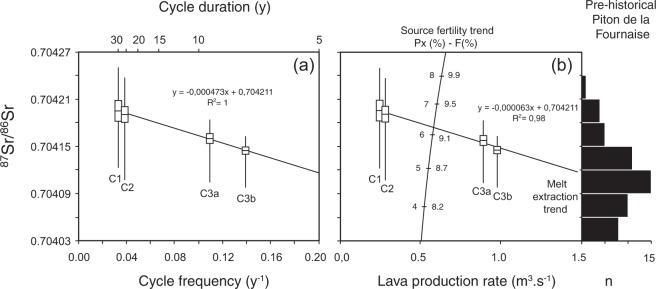
Correlation between ^87^Sr/^86^Sr, cycle duration (**a**) and lava production rate (**b**). (**a** and **b**) Average ^87^Sr/^86^Sr (with boxes for standard errors) and ^87^Sr/^86^Sr range (bar) are reported for the completed eruptive cycles C1, C2, C3a and C3b. (**b**) The trend accounting for source fertility is drawn assuming that lava production rate is proportional to the degree of partial melting. Numbers along the trend refer to the fraction of G2-type pyroxenite (Px %) in the melting source and the corresponding bulk degree of melting (F %) (see methods). The overall decrease of ^87^Sr/^86^Sr with increasing lava production rate is ascribed to the efficiency of melt extraction from the peridotite (trend labelled “melt extraction”). The histogram indicates the distribution of ^87^Sr/^86^Sr in the 40–530 ky old lavas from Piton de la Fournaise^22^.

In addition to the cyclic variations, the average ^87^Sr/^86^Sr shows an overall decrease from cycle 1 to cycle 3b that correlates with a decrease of cycle duration (from 30 to 7 years), a fourfold increase of lava production rate (from 0.25 to 0.97 m^3^/s), and thus presumably a comparable increase of magma supply rate (Fig. 2). This trend is also accompanied by a decrease in ^87^Sr/^86^Sr variability. The negative correlation between ^87^Sr/^86^Sr and lava eruption rate suggests that melting of mafic veins during cycles 1 and 2 subsequently facilitated melt extraction from the peridotite matrix and ultimately resulted in higher production of more depleted lava during cycles 3a and 3b. Although the times-scale is longer than that of individual cycles, the process seems to be fundamentally the same. This suggests some kind of fractal distribution of fertile veins in the mantle source, and a melt network resembling a fractal tree^31^. Our inference regarding the depleted signature and high productivity of cycle 3 is supported by the model of reactive flow, initially proposed for melt extraction beneath mid-ocean ridges^32,33^. In this model, enriched melts produced by deep melting of mafic veins, initially Si-rich, become silica-undersaturated as they ascend. In the upper melting column (above the garnet-spinel transition), such melts dissolve orthopyroxene and precipitate olivine, forming high porosity dunite channels that subsequently drain low porosity melts from the surrounding peridotite and allow fast melt transport through the lithosphere^32,33^. By promoting melt extraction, reactive flow of enriched melts results in more depleted residual sources, as observed along mid-ocean ridges in the vicinity of hotspots^33^. At Piton de la Fournaise, an unexpected, indirect consequence of source fertility is the higher production of more depleted lavas. The rapid evolution of lava composition and eruption rate from cycle 1 to cycle 3b is consistent with the short lifetime of dunite conduit^34^. Frequent eruption of olivine-rich lavas during cycle 3 (with 8 eruptions between 2001 and 2007), compared to cycle 1 (3 eruptions) and cycle 2 (3 eruptions) could also result from flushing out dunite conduits. Conversely, the small variations of ^230^Th-^238^U and ^226^Ra-^230^Th disequilibria in historical lavas of Piton de la Fournaise^17,35^, and their poor correlation with ^87^Sr/^86^Sr, do not support the reactive flow model, and additional modelling is required to evaluate this issue.

## Discussion

### Broader implications for Piton de la Fournaise and Kilauea volcanoes

Although pyroxenite is probably a minor lithology of the Reunion mantle plume^23,26,27^, its behaviour during melting has a twofold control on magma supply, and ultimately on the short-term eruptive activity of the Piton de la Fournaise volcano. First, melting of fertile veins seems to trigger melt extraction, so that the distribution of veins in the melting region directly influences the timing of magma supply. However, melting of a slightly more fertile source has a small influence on the melt production rate because the bulk extent of melting barely increases (Fig. 1a). Second, melting of fertile veins promotes melts channelization and melts extraction from the peridotitic matrix, increasing the volume from which melt is extracted and ultimately the rate of magma supply, while the bulk extent of melting slightly decreases (Fig. 2b). On the geochemical standpoint, the small variations in the bulk extent of melting preserve the positive correlation between ^87^Sr/^86^Sr and ratios of highly to moderately incompatible trace elements inherited from the source (Supplementary Fig. S3), despite the large extent of melting of the pyroxenite (17% and 50% for KG-1 and G2 compositions, respectively). Noteworthy, an unexpected, indirect consequence of melting fertile veins is thus to decrease the ^87^Sr/^86^Sr ratios of lavas (melt extraction trend labelled in Fig. 2b).

The ^87^Sr/^86^Sr ratio of lavas erupted between 40 and 530 ky ago at Piton de la Fournaise shows high frequency fluctuations within a range (0,704034–0,704227)^22^ that is very similar to the modern range, suggesting that Piton de la Fournaise pre-historic eruptive regime was to a first order similar to that observed today. Closer inspection of data reveals that modern lavas have on average slightly more radiogenic ^87^Sr/^86^Sr (0.704172 ± 3.10^−6^ (SE), n = 139) compared to prehistoric lavas (0.704112 ± 7.10^−6^ (SE), n = 55). The slight increase in ^87^Sr/^86^Sr correlates with the eruption of less differentiated magma through time^22^, and presumably with an increase in the rate of magma supply, suggesting that Piton de la Fournaise activity has increased through time in response to tapping a more fertile source. This long-term evolution could be related to the upwelling of more fertile material through the melting region. Conversely, the factor of four increase of eruption rate (from 0,25 to 0,97 m^3^/s) during the most recent period (1998–2014) is a transient phenomenon possibly resulting from melts channelization, which occurred following reactive flow of enriched melts between 1942 and 1992.

Compared to Piton de la Fournaise, the historical activity of Kilauea volcano is characterized by both longer eruptions (e.g., sustained lava lake at the summit from 1823 to 1924, 1969–1974 Mauna Ulu, and Pu’u ‘Ō‘ō eruption that lasted for 35 years), and longer repose periods (e.g., 1934–1952)^36,37^. Such difference is consistent with the occurrence in the Hawaiian plume of compositional heterogeneities that are one order of magnitude larger^10,38^ than those that we estimate in the Reunion plume (0.06–0.16 km^3^). Over the last two centuries, the eruption rate of Kilauea decreased by a factor of ten between 1880 and 1960^7^. This correlates with a decrease of the degree of partial melting and ^87^Sr/^86^Sr^7^, in keeping with the melting of a less fertile source^8^. At shorter time-scale, the Pu’u ‘Ō‘ō eruption was increasingly fed during the first 20 years by chemically depleted but high- ^87^Sr/^86^Sr melts^30^, until the ^87^Sr/^86^Sr temporal trend reversed during the 2003–2007 magma surge^10^. The decrease of ^87^Sr/^86^Sr during the 2003–2007 magma surge may be compared to that observed during the recent eruptions of Piton de la Fournaise. In both cases, the transient increase in lava eruption rate might be the result of fast transport of melts within high-porosity dunite channels that were formed by reactive flow of enriched melts during earlier melting events. This model still needs to be more thoroughly tested, at Reunion and at Hawaii.

## Methods

### Sampling

Lava flows emplaced before 1975 were sampled several years after each eruptions, and only one sample per eruption has generally been analysed. In these cases, the exact sample eruption dates are not well constrained. Since 1975, lavas were collected during or shortly after each eruption, so the eruption dates of the samples are precisely known. Following the creation of the permanent volcanological observatory (OVPF) in 1980, eruptions were sampled continuously allowing the resolution of compositional variations within individual events. The sampling and the analyses are now reinforced by the contribution of the observation system, DynVolc^39^, led by LMV and OVPF.

### Geochemical analysis

All new analyses reported in Supplementary Table S2 were acquired at the LMV (Clermont-Ferrand, France). Samples were crushed into millimetre -size chips using home–made thermally hardened steel jaws, and powdered in a motorised agate mortar. Major elements were analysed by Inductively Coupled Plasma Optical Emission Spectrometry (ICP-OES, HORIBA Jobin Yvon Ultima C) following a Lithium metaborate (LiBO_2_) fusion method, while trace elements were analysed using a Quadrupole Mass Spectrometer (ICP-MS, Agilent 7500) following acid dissolution (HF-HNO_3_) of rocks in teflon vials. The external precision of major and trace element analysis, inferred from the repeated analysis of the BHVO-2 standard, is better than 10% (2σ), except for Pb (20%). Strontium and Pb were purified using Eichrom specific resins (Sr.Spec), and their isotopic compositions were measured by Thermal Ionisation Mass Spectrometry (TIMS Thermo Triton) and Inductively Coupled Plasma Multi-collector Mass-Spectrometer (MC-ICP-MS Thermo Neptune plus), respectively. The precisions (2σ error) of Sr and Pb isotope measurements are 15 and 50 ppm/amu, respectively. A detailed description of the methods of element separation and isotope ratios measurement is given in Vlastelic *et al*.^21^.

### ^87^Sr/^86^Sr melt extraction trajectories (MET)

The Reunion mantle plume is assumed to be made of small pyroxenite veins embedded in a peridotitic media^40^. Reunion submarine pre-shield lavas (^87^Sr/^86^Sr up to 0.7048), which sampled the most fertile region of the plume^41^, and Mauritius post-shield lavas (average ^87^Sr/^86^Sr of 0.7038), which sampled plume matrix^40^, are used for pyroxenite and peridotite isotope compositions, respectively. For the purpose of modelling melt extraction trajectories, we use a simple model where pyroxenite and peridotite lithologies melt independently (no reaction)^28^. The compositions of instantaneous melt and residue, and that of pooled melts are calculated every 0.05 GPa pressure increment using the batch melting equation. Above the peridotite solidus, pooled melts from the pyroxenite and peridotite lithologies are mixed according to their mass proportion. Both lithologies have solidus slopes of 130 °C/GPa. We consider a mantle plume with a potential temperature of 1450 °C (ca. 150 °C excess temperature) crossing the peridotite solidus at 3.1 GPa. Melting ends at the lithosphere-asthenosphere boundary located near 2.4 GPa^42^. Highly (G2- type) and moderately (KG-1 type) fertile pyroxenites starting to melt 1.3 and 0.1 GPa deeper than the peridotite, respectively, are considered^43–45^. KG-1 is made of equal amounts of G2 and KLB-1 peridotite, and can be considered as a fertile peridotite^43^. Following Stracke and Bourdon^28^, we assume that the melting productivity of pyroxenite increases linearly from 15 to 70%/GPa until the start of peridotite melting, and is constant at 16.5%/GPa thereafter. Melting productivity of peridotite increases with decreasing depth of melting from 6.6 to 67%/GPa following a power law. In such configuration, the extent metling of the peridotite is 6.5% and that of the pyroxenite ranges from 17% (KG-1 type) to 50% (G2- type). Pyroxenite and peridotite Sr concentrations are assumed to be 78,3 ppm (recycled MORB) and 9,8 ppm (average DMM), respectively. Pyroxenite/melt and peridotite/melt Sr partition coefficients are assumed to be 0,05 (average pyroxenite) and 0,03 (garnet peridotite).

### Volumes of the pyroxenitic and peridotitic mantle sources

The volume of mantle melt (V_melt_) is derived from the volume of lava (V_lava_) through three corrections. First, the dense rock equivalent volume (V_DRE_) is inferred from:

1 $${{\rm{V}}}_{{\rm{DRE}}}=(1-{\rm{\varphi }}){{\rm{V}}}_{{\rm{lava}}}$$

where ϕ is lava porosity, close to 50% on average for lava flows^46^. Then, the volume corrected for olivine fractionation and/or accumulation (V_12.5_) is obtained by normalizing V_DRE_ to primitive melt MgO content of 12.5 wt%^25^:

2 $${{\rm{V}}}_{12.5}={{\rm{V}}}_{{\rm{DRE}}}(1+(12.5{{\rm{MgO}}}_{{\rm{sample}}})/({{\rm{MgO}}}_{{\rm{olivine}}}-12.5))$$

where olivine MgO content is 43 wt% on average^22^. The volume of mantle melt is derived by considering that all mantle melt produced is not extruded:

3 $${{\rm{V}}}_{{\rm{melt}}}={{\rm{V}}}_{12.5}/(1-{\rm{i}})$$

where i is the intrusion rate, in the range of 10–20%^36^.

The volumes of pyroxenite and peridotite melts are inferred from the mass fractions assuming, in a first approximation, identical density for pyroxenite and peridotite melts:

4 $${{\rm{V}}}_{{\rm{pyr}}}={{\rm{x}}}_{{\rm{pyr}}}{{\rm{V}}}_{{\rm{melt}}}({\rm{and}}\,{{\rm{V}}}_{{\rm{per}}}={{\rm{x}}}_{{\rm{per}}}{{\rm{V}}}_{{\rm{melt}}})$$

where the mass fraction of pyroxenite and peridotite melts (x_pyr_ and x_per_, respectively) are derived from Sr isotope mass balance:

$$\begin{array}{c}{{\rm{x}}}_{{\rm{pyr}}}\cdot {{\rm{Sr}}}_{{\rm{pyr}}}\cdot ({({}^{87}{\rm{Sr}}/{}^{86}{\rm{Sr}})}_{{\rm{sample}}}-{({}^{87}{\rm{S}}{\rm{r}}/{}^{86}{\rm{S}}{\rm{r}})}_{{\rm{pyr}}})+\\ \,{{\rm{x}}}_{{\rm{per}}}\cdot {{\rm{Sr}}}_{{\rm{per}}}\cdot ({({}^{87}{\rm{Sr}}/{}^{86}{\rm{Sr}})}_{{\rm{sample}}}-{({}^{87}{\rm{Sr}}/{}^{86}{\rm{Sr}})}_{{\rm{per}}})=0\end{array}$$

with

5 $$\,{{\rm{x}}}_{{\rm{pyr}}}+{{\rm{x}}}_{{\rm{per}}}=1$$

Following the MET model described above, (^87^Sr/^86^Sr)_pyr_ = 0.7048 and (^87^Sr/^86^Sr)_per_ = 0.7038, and Sr_pyr_/Sr_per_ = 1.21 in pooled melts (model result with G2 configuration).

Because x_pyr_ and x_per_ vary within eruption cycles, the volume of each component is integrated sample by sample by combining Eqs 1–5. Lastly, the volumes of the pyroxenitic and peridotitic mantle sources are derived from the volumes of melt using the melting extents of 50 and 6.5%, respectively (model results with G2 configuration).

## Electronic supplementary material

Table S1

Table S2

Figure S3

## Data Availability

All data generated during this study are included in this published article (and its Supplementary Information files).
