# Supplementary figures and images for: Control of source fertility on the eruptive activity of Piton de la Fournaise volcano, La Réunion
Source: Sci Rep. 2018 Sep 27;8:14478. doi: 10.1038/s41598-018-32809-0 (PMC6160422; doi:10.1038/s41598-018-32809-0)

Supplementary Figure S3 :  $^{87}\text{Sr}/^{86}\text{Sr}$  versus Th/Yb in historical lavas of Piton de la Fournaise.

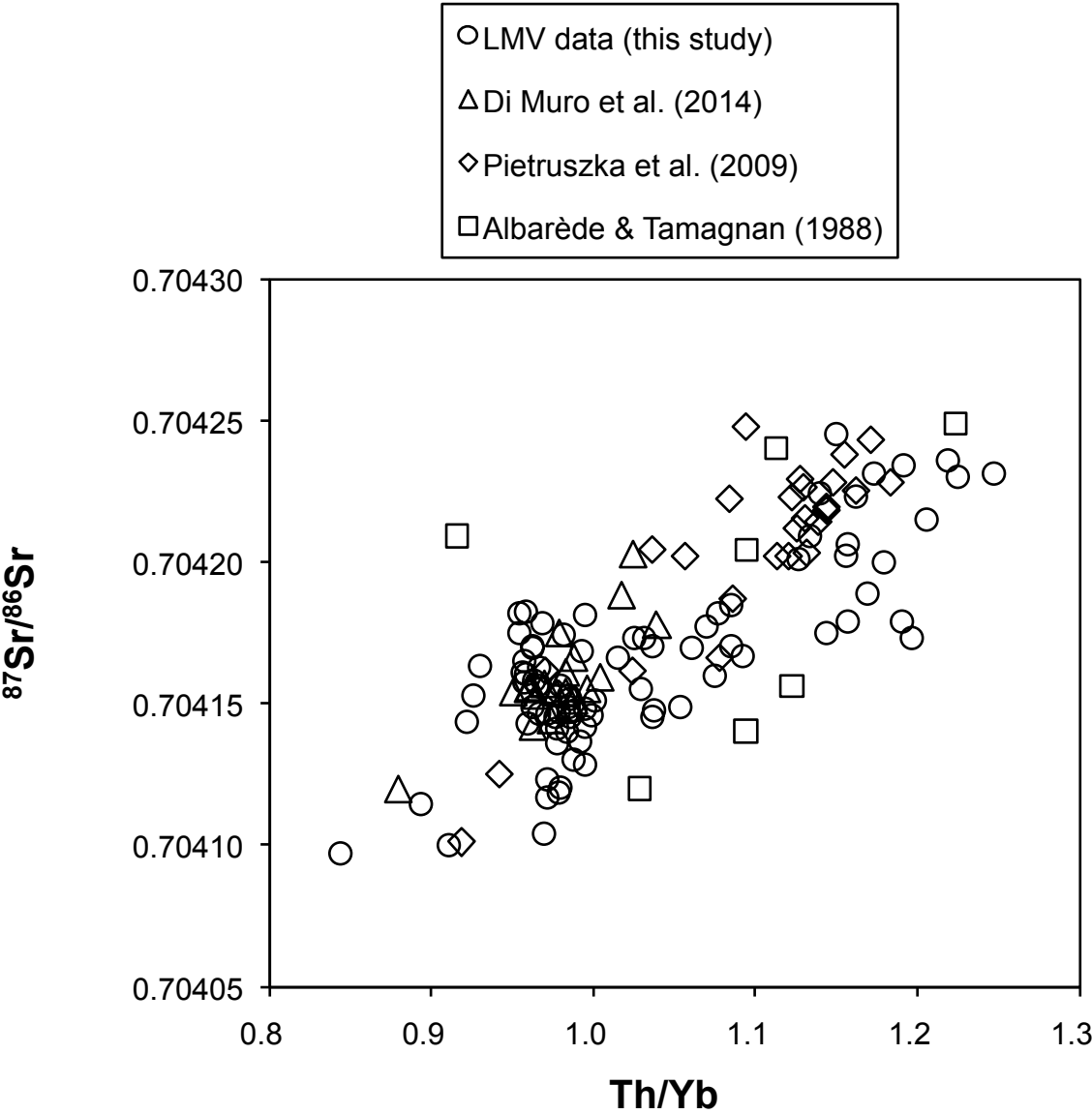

Supplement: Supplementary file 3 — Figure S3 [file 41598_2018_32809_MOESM3_ESM.pdf]
